# The Effects of Claw Health and Bone Mineral Density on Lameness in Duroc Boars

**DOI:** 10.3390/ani13091502

**Published:** 2023-04-28

**Authors:** Jinxin Lu, Lingling Hu, Liangliang Guo, Jian Peng, Yinghui Wu

**Affiliations:** 1 Department of Animal Nutrition and Feed Science, College of Animal Science and Technology, Huazhong Agricultural University, Wuhan 430070, China; 2 The Cooperative Innovation Center for Sustainable Pig Production, Wuhan 430070, China

**Keywords:** claw lesion types, bone mineral density, lameness, Duroc boars

## Abstract

**Simple Summary:**

Lameness shortens the longevity and decreases the production efficiency of commercial herds. However, little research has been conducted on its causes in boars. In this study, we evaluated the prevalence of lameness from two aspects, claw health and bone health, using them for the first time to predict lameness in boars. The results show that claw lesions are widespread in boar populations, and swelling ankle (SWE) is significantly associated with lameness. In addition, lameness affected 22.86% of the boars in the osteopenia group. The prevalence of lameness was significantly higher in boars with osteopenia. Further analysis revealed that boar bone mineral density (BMD) was corelated to age, housing types, and serum Ca. Age had a convex quadratic curve relationship with bone mineral density, and the highest value was observed at the age of 43 months. More importantly, studies show that the bone mineral density of boars in individual pens was significantly higher than that of boars in individual stalls. Therefore, boars with different bone mineral density in different housing types may serve as a guide to improve boar lameness. It is necessary to improve the claw and bone health of boars in production to reduce the occurrence of lameness and improve the breeding value of boars.

**Abstract:**

To investigate the effects of claw lesion types and bone mineral density on lameness in boars, the data of claw lesion score, gait score, and bone mineral density, measured by a Miniomin ultrasound bone densitometer, were collected from a total of 739 Duroc boars. Firstly, we discovered that the prevalence of claw lesions was as high as 95.26% in boars. The percentage of lameness of boars with SWE was higher than those with other claw lesions. Meanwhile, the results showed that the probability of lameness was higher in boars with lower bone mineral density (*p* < 0.05). Logistic regression models, including variables of boar age, body weight, serum mineral level, and housing type, were used to identify the influencing factors of bone mineral density in this study. The results found that bone mineral density increases with age before reaching a maximum value at 43 months of age, and begins to decrease after 43 months of age. Elevated serum Ca levels were significantly associated with an increase in bone mineral density (*p* < 0.05). Aside from the above findings, we also made an interesting discovery that boars in the individual pen model significantly increased bone mineral density compared to those in the individual stall model. In conclusion, claw lesions and bone mineral density were significantly associated with lameness. Age, serum Ca, and housing type are the potential influencing factors for bone mineral density in boars.

## 1. Introduction

In pig production, with the fast development of large-scale farming, lameness has become an increasingly great concern, being an important factor limiting pig production [1,2]. Moreover, lameness is one of the main reasons for culling [3], which shortens the herd life expectancy of pigs [4,5] and causes huge economic losses to the pig industry worldwide [6]. However, many studies have reported the effect of lameness on sow, but few studies have been reported on boars. Differences in feeding mode and uses between boars and sows may result in the formation of lameness in boars not being the same as in sows. The premature culling of boars reduces sperm production and the number of offspring which they can provide over their lifetime [7]. Therefore, it is of great significance to study lameness in boars.

Claw lesions, osteoarthritis, and osteochondrosis are listed as the top three causes of lameness in pigs [8]. Studies have shown that the morbidity rate of claw lesions in sows was above 50%, which has become a severe problem that cannot be ignored. In general, minor injuries do not affect the movement of pigs, but the increasing severity of claw lesions makes pigs susceptible to the development of lameness [9]. However, the effect of claw lesion types on lameness, such as white line (WL), toes (TOE), cracked wall vertical (CWV), heel overgrowth and erosion (HOE), dew claws (DEW), heel sole crack (HSC), swelling ankle (SWE), and cracked wall horizontal (CWH), has not yet been studied extensively.

Bone mineral density is widely accepted as the gold standard for determining bone mass in clinical practice [10]. Increased bone resorption and/or decreased bone formation can reduce bone mineral density, leading to osteopenia, which is also reflected by the serum turnover markers [11]. Decreased bone mineral density can lead to bone diseases related to loss of bone mass, such as osteoporosis [12]. Lameness has been determined using several methods, including claw lesion score and gait score [13]. However, these methods are subjective and cannot accurately reflect the skeletal problems of boars. Therefore, a more precise method should be applied to reflect lameness so that it can be better improved. Dual-energy X-rays (DEX) and ultrasound are the most common clinical techniques for measuring bone density. Compared to ultrasound, DEX may be more accurate but involves radiation exposure. As a result, it is not recommended for children or pregnant women. On the other hand, ultrasound does not involve radiation and is more portable and convenient to use [14]. Bone mineral density is defined as the amount of minerals per unit of bone volume and can reflect bone health. Some studies have shown that bone mineral density can accurately predict fracture risk [15]. However, no such studies have been conducted on the relationship of bone mineral density with lameness in breeding boars. Therefore, bone mineral density could be an additional tool to predict lameness in the future.

There are many other factors that could affect bone mineral density, such as age, exercise, body weight, and serum mineral level [16]. As we all know, age can significantly affect bone mineral density. During a person’s lifetime, bone mineral density increases with increasing age; after reaching its maximum weight around 30~40 years old, bone mineral density begins to decrease with advancing age [17]. Exercise plays an important role in the whole lifespan of mammals, and it has been found to increase bone mineral density and bone strength in sows [18]. Minerals are inorganic components of bone, which are important for maintaining bone health [19]. Lameness during rearing and lactation was decreased by supplementing organic trace minerals [20]. Identifying factors that influence bone mineral density might decrease the prevalence of lameness and lead to an increase in boar fertility in commercial herds. However, the effects of the above influencing factors on bone mineral density in boars have not been reported.

In our study, we aimed to explore the potential relationship between claw lesion types, bone mineral density, and lameness. At the same time, we wanted to explore the risk factors affecting bone mineral density in the aspects of body weight, age, and housing types, as well as serum Cu, Ca, P, Zn, Mg, Fe, Mn, Se, Pb, and Cd by using the multivariate ordered logistic regression model in commercial herds.

## 2. Materials and Methods

### 2.1. Experimental Design

This study was performed from November 2021 to April 2022 at an artificial insemination center located in Southern China. A total of 739 Duroc boars aged 10 to 67 months with an average age of 22 months were selected. The experimental boars were placed within individual pens (IP) (3.0 × 2.5 m^2^) and individual stalls (IS) (2.43 × 0.73 m^2^) that have a slatted concrete floor. There were 375 boars in the IP model and 364 in the IS model. An automated production system, which included a positive pressure ventilation, automatic feeding, and heating systems, was used to control the indoor environment (Automated Production Systems, 1004 E. Illinois St. Assumption, IL 62510, USA).

### 2.2. Evaluation Methods

To examine claw lesions of boars, we followed the previously reported protocol [13]. The experiments were performed during the night while the boars rested. In this study, to minimize variability, all measurements were made by the same person. The lesions observed included WL, TOE, CWV, HOE, DEW, HSC, SWE, and CWH. The lesions of the front and hind feet were defined from 0 to 3 ranging from normal to severe lesions.

To evaluate the lameness of boars, a scoring system from 0 (not lame) to 5 (severely lame) was used [21]. Scoring was based on the gait exhibited by the boar as it moved. As in our previous study, a new classification criterion that used two categories—normal (0–2) and lameness (3–5)—was used in this study to determine whether the boars were lame [22]. The evaluation standard of claw lesions is presented in Table 1.

### 2.3. Element Determination in Serum Samples

Serum samples from 739 boars were collected to determine the concentrations of 10 elements, including Ca, P, Mg, Cu, Fe, Zn, Mn, Se, Pb, and Cd, by using inductively coupled plasma mass spectrometry (Agilent 7900, Agilent Technologies, Tokyo, Japan) [23]. The Porcine CTX-I (CTX-I) ELISA Kit and the Porcine Bone gla protein; Osteocalcin (BGP; OCN) ELISA Kit were used to measure serum markers of pig bone turnover such as osteocalcin (OCN) and C-terminal telopeptide of type I collagen (CTX-1) (Shanghai mlbio Biotechnology Co., Ltd., Shanghai, China). All of the tests were carried out according to the manufacturers’ instructions. After blood collection, serum samples were obtained by centrifugation at 1500× *g* for 10 min at room temperature. Serum samples were stored at −80 °C until trace element analysis.

### 2.4. Measurement and Classification of Bone Mineral Density

Bone densitometry was performed on the medial metatarsal bone of the left hind limbs of boars using a Sunlight MiniOmni Ultrasound Bone Densitometer (Sunlight Medical Ltd., Tel Aviv, Israel), and the velocity of ultrasound propagation through the bone was measured as the speed of sound (SOS, m/s). Previous studies have shown that SOS is positively associated with bone mineral density [24,25]. SOS, which is widely used clinically, has been used to characterize bone mineral density [26]. Bone mineral density was graded according to the recommendations of the World Health Organization in 1994. Strong bone was defined as 1 standard deviation (SD) above the mean SOS value ($\bar{X}$) of boars; the mean SOS value of boars plus or minus 1 SD was defined as normal bone; osteopenia was defined as 1 to 2.5 SDs below the mean SOS value of boars; osteoporosis was defined as 2.5 SDs below the mean SOS value of boars [27].

In order to ensure sufficient sample size in each group, bone mineral density was classified as follows: grade 1, SOS ≥ 4426 m/s; grade 2, 3976 m/s ≤ SOS ≤ 4425 m/s; grade 3, SOS < 3975 m/s. The data distribution of bone mineral density is shown below (Table 2).

### 2.5. Statistical Analysis

All the statistical procedures were performed with SPSS (SPSS for Windows, version 20.0, Chicago, IL, USA).

Crosstab analysis was used to calculate the contingency correlation (Φ) between lameness and claw lesions. In the crosstab analysis, both the dependent and independent variables are binary variables; lameness and claw lesions were not considered to be associated if Φ < 0.2.

The impact of different claw lesions, housing type, and bone mineral density on lameness was tested using a chi-square test. Post-hoc multiple comparisons were carried out after choosing the Bonferroni correction *p*-value in a contingency table.

Data from two groups were analyzed by the *t*-test and data from more than two groups were analyzed by one-way ANOVA. Post-hoc multiple comparisons were carried out after choosing the Tukey–Kramer test. For all comparisons, differences were considered significant if *p* < 0.05.

Univariate analysis was performed on the independent variables. The independent variables with *p*-values < 0.1 in the univariate analysis were included in the multivariate logistic regression, and the multivariate ordered logistic regression model was utilized to identify the potential risk factors using the forward stepwise selection method with *p* < 0.05. The model is formulated as follows:

Logit (P) = β0 + β1 A + β2 B + β3 C + β4 D + β5 E + β6 F

β0 is the intercept. A, B, C, D, E, and F represent housing type, age, body weight, serum Ca, serum P, and serum Zn, respectively. β1 (includes 2 dummy variables), β2 (includes 4 dummy variables), β3 (includes 4 dummy variables), β4 (includes 3 dummy variables), β5 (includes 3 dummy variables), and β6 (includes 3 dummy variables) are the slope for each dummy variable.

## 3. Results

### 3.1. The Effect of Claw Lesion Types on Lameness

**Figure 1:**
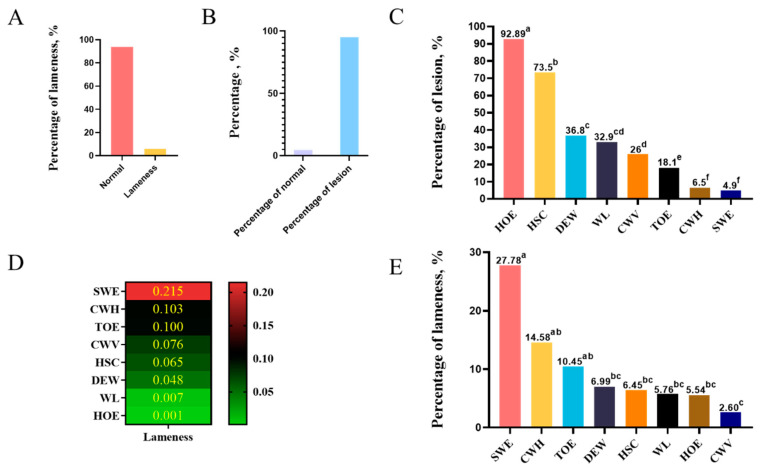
The effect of claw lesion types on lameness. (**A**) Percentage of Duroc boars with lameness and normal boars. (**B**) Percentage of claw lesions and normal claws in Duroc boars. (**C**) Percentage of different claw lesion types in Duroc boars. (**D**) Heat map of correlation between different claw lesion types and lameness. *p*-values for SWE, CWH, TOE, and CWV were less than 0.05. (**E**) The prevalence of lameness with different claw lesion types. Different lowercase letters indicate statistically significant differences in the prevalence of lameness among different claw lesions.

To investigate the relationship between claw lesion types and lameness, we used data collected from 739 Duroc boars, which include lameness and claw lesion types. First, we found a low percentage of boars suffering from lameness, probably because boars are culled if they have severe lameness (Figure 1A). However, the percentage of claw lesions was 95.26% in the studied population, whereas the proportion of normal boar was only about 4.74% (Figure 1B). The prevalence of different claw lesion types in boars from high to low was HOE, HSC, DC, WL, CWV, TOE, CWH, and SWE. Moreover, HOE and HSC were more prevalent than other types (Figure 1C). Furthermore, we found a substantial association between SWE and lameness through the crosstab analysis (*p* < 0.001) (Figure 1D). The prevalence of lameness with different claw lesion types is summarized in Figure 1E. The results show that all claw lesion types can influence the prevalence of lameness. Boars with SWE had the highest percentage of lameness.

### 3.2. The Relationship between Bone Mineral Density and Lameness

**Figure 2:**
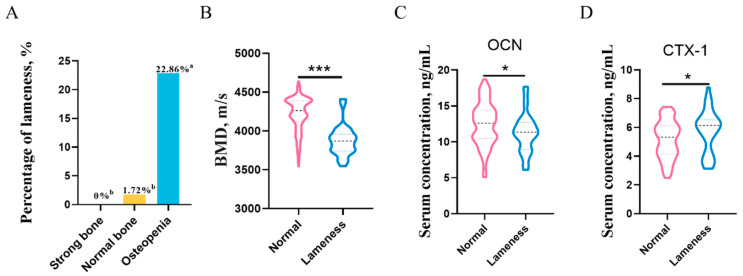
The relationship between bone mineral density and lameness. (**A**) The prevalence of lameness with different bone mineral density. (**B**) The bone mineral density in lame and non-lame Duroc boars. (**C**) The bone formation marker OCN in lame and non-lame Duroc boars. (**D**) The bone resorption marker CTX-1 in lame and non-lame Duroc boars. Different lowercase letters indicate significant differences in the prevalence of lameness among different bone mineral density. * Significant at the level *p* < 0.05, *** significant at the level *p* < 0.001.

Interestingly, the findings show that the prevalence of lameness in boars with osteopenia was significantly higher than in other groups (*p* < 0.05) (Figure 2A). Specifically, compared to normal boars, boars with lameness showed significantly lower bone mineral density (*p* < 0.001) (Figure 2B). Consistent with the data of bone mineral density, boars with lameness showed significantly lower levels of bone formation marker OCN and higher levels of bone resorption marker CTX-1 than normal boars (*p* < 0.05) (Figure 2C,D).

### 3.3. Factors Affecting the Bone Mineral Density

#### 3.3.1. Potential Risk Factors of Bone Mineral Density

To better understand the potential risk factor of bone mineral density in boars, univariate logistic regression analysis was performed to analyze the influence of body weight, age, and housing type, as well as serum Cu, Ca, P, Zn, Mg, Fe, Mn, Se, Pb, and Cd on bone mineral density. Based on the results of the univariate logistic regression analysis, independent variables with *p* < 0.10 were included in the multivariate ordered logistic regression model so as not to omit the potential influencing variables. The results showed that bone mineral density was influenced by age, body weight, housing type, and serum Ca and P (Table 3).

#### 3.3.2. The Factors Affecting Bone Mineral Density Were Screened by Using the Multivariate Ordered Logistic Regression Model

The results of the multivariate ordered logistic regression model analysis of the selected factors in the bone mineral density model are summarized in Table 4. The bone mineral density was significantly influenced by housing type, age, and serum Ca in the study (*p* < 0.05). Specifically, boars housed in the IS model had lower bone mineral density than boars housed in the IP model (*p* < 0.001). Boars aged younger than 12 months, 13–24 months, and 25–36 months had lower bone mineral density than boars aged older than 37 months (*p* < 0.05). Meanwhile, boars with serum Ca ≤ 8 mg/dL had lower bone mineral density than those with serum Ca ≥ 11 mg/L (*p* = 0.034). We found that serum P had no statistically significant effect on bone mineral density. The effect of serum P on bone mineral density may be masked by serum Ca.

### 3.4. The Effect of Factors on Bone Mineral Density in Duroc Boars

#### 3.4.1. The Effect of Age on Bone Mineral Density in Duroc Boars

**Figure 3:**
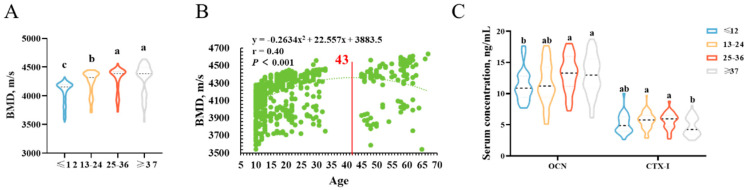
The effect of age on bone mineral density in Duroc boars. (**A**) Change in bone mineral density as a function of age in Duroc boars. (**B**) Relationships between age and bone mineral density. (**C**) Changes in bone turnover marker levels with advanced age in Duroc boars. Different lowercase letters indicate significant differences in BMD among different age groups.

According to the variations in fecundity with age, the boars were divided into four groups. The results found that there is an increase in bone mineral density with advanced age (*p* < 0.05). After 37 months of age, there is a continued tendency for an increase in bone mineral density (Figure 3A). Statistical analyses revealed a highly significant effect of age on bone mineral density by applying a second-order equation (*p* < 0.001). The maximum bone mineral density is reached at 43 months of age (Figure 3B). Consistent with previous findings, OCN, a marker of bone formation, increased with advanced age, and CTX-1, a marker of bone resorption, increased first and then decreased at the end (Figure 3C).

#### 3.4.2. The Effect of Housing Type on Bone Mineral Density in Duroc Boars

**Figure 4:**
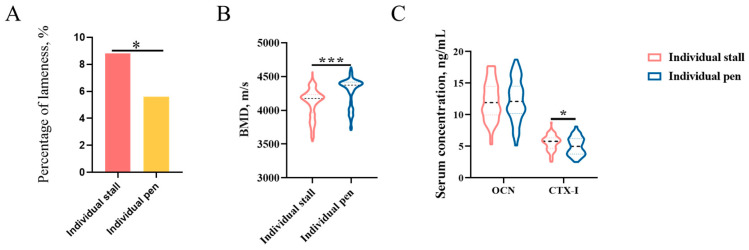
The effect of housing type on bone mineral density in Duroc boars. (**A**) Effect of housing type on prevalence of lameness in Duroc boars. (**B**) Effect of housing type on bone mineral density in Duroc boars. (**C**) Effect of housing type on bone turnover marker levels in Duroc boars. * Significant at the level *p* < 0.05, *** significant at the level *p* < 0.001.

Compared with the large individual pen model, boars have a significantly higher prevalence of lameness and lower bone mineral density in the IS model (Figure 4A,B). There is no difference in the bone formation marker OCN between the IS model and the large individual pen model. However, the bone resorption marker CTX-1 in the IS model was significantly increased compared to the large individual pen model (Figure 4C).

#### 3.4.3. The Effect of Serum Ca and Serum P on Bone Mineral Density in Duroc Boars

**Figure 5:**
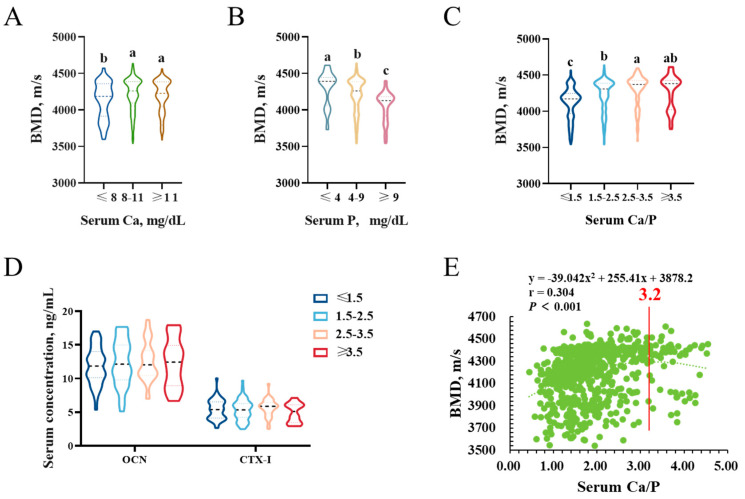
The effect of serum Ca and serum P on bone mineral density in Duroc boars. (**A**) Effect of serum Ca on bone mineral density in Duroc boars. (**B**) Effect of serum P on bone mineral density in Duroc boars. (**C**) Effect of calcium-to-phosphorus ratio on bone mineral density in Duroc boars. (**D**) Effect of calcium −to−phosphorus ratio on bone turnover marker levels in Duroc boars. (**E**) Relationships between calcium-to-phosphorus ratio and bone mineral density. Different lowercase letters indicate significant differences between different groups at *p* < 0.05.

As shown in Figure 5A, when serum Ca concentration was 8–11 mg/dL in boars, bone mineral density was significantly increased compared to those with serum Ca ≤ 8 mg/dL (*p* < 0.05). Nevertheless, when serum Ca exceeds 11 mg/dL, bone mineral density no longer increases. Unexpectedly, elevated serum P levels were significantly associated with a decrease in bone mineral density (Figure 5B). The relationship between the calcium-to-phosphorus ratio and bone mineral density is presented in Figure 5C. Statistical analyses revealed a highly significant effect of the calcium-to-phosphorus ratio on bone mineral density by applying a second-order equation (*p* < 0.001). However, the calcium-to-phosphorus ratio had no significant effect on serum biochemical markers of bone turnover (Figure 5D). Bone mineral density reached its maximum when the calcium-to-phosphorus ratio was 3.2 (Figure 5E).

## 4. Discussion

Boar breeding, although accounting for a small percentage of the pig population (approximately 2–5%), is critical to the whole pig industry chain [28,29]. This study was the first investigate the influence of claw lesion types and bone mineral density on lameness in Duroc boars, which is one of the key indicators of animal welfare and economic benefits in pig production [2].

Claw lesions are one of the top three leading causes of lameness in pig production [2]. When pigs suffer from claw lesions, slight damage to the hoof does not cause significant pain, but as the degree of damage intensifies, the pig may exhibit lameness [1]. Lameness is one of the leading reasons for the early culling of breeding pigs [6]. As reported by a few previous studies, the most common reasons for the removal of boars were lameness (36%), poor semen quality (28%), death (6%), and old age (5%) [3]. Consequently, extending boar herd life expectancy by reducing the prevalence of lameness is an important way to improve the productivity of pig farms. Studies about the impact of claw lesion types on lameness have been more frequently reported for sows, but data on lameness in boar are scarce. In this work, we found that the rate of claw lesions is as high as 95.26%. The possibility that SWE can affect lameness is far greater than other claw lesions. This result was similar to the observations of previous studies, which found a moderately significant positive correlation between boar lameness and swelling ankle (Φ = 0.5571) [13]. At the same time, it is worth noting that the main causes of SWE are wet and dirty floors [9]. Accordingly, dry and clean floors may help to reduce the development of SWE.

Previous studies on lameness mainly focused on the effects of claw lesion score and lameness scores [13]. In this paper, we have introduced bone mineral density to predict lameness in boars for the first time. Bone mineral density is a surrogate indicator directly related to bone mass and is widely used to monitor and diagnose the health status of bone in clinical practice [30,31]. However, to the best of our knowledge, no prior studies have reported on the relationship between lameness and bone mineral density in boars. For the first time, we discovered that the bone mineral density of Duroc boars was associated with lameness. Previous research has shown that the heritability of the strength of limbs of pigs is approximately 0.1–0.5 [32]. Therefore, detecting the bone mineral density of boars can provide a new way to predict lameness. However, a causal relationship between lameness and BMD could not be demonstrated in our experiments, so further experiments are needed to verify it. Nevertheless, it makes sense to select pigs with better bone quality by measuring bone mineral density, thus reducing the occurrence of lameness and prolonging the herd life expectancy of boars.

Bone mineral density of boars was measured by using a portable broadband ultrasound bone densitometer in our study. We found that boars with osteopenia had a significantly higher rate of lameness than boars with strong and normal bone. This is an extremely interesting discovery as this work for the first time revealed a link between bone mineral density and lameness. Therefore, we explored the difference in bone mineral density between lame and non-lame boars. Interestingly, we found that the bone mineral density of lame boars is significantly lower than that of non-lame boars. Furthermore, in lame boars, higher resorption markers and lower bone formation markers were observed compared to non-lame boars. These symptoms are similar to bedridden patients [33]. This finding is meaningful since it provides a possible way to enhance bone quality and predict lameness.

In order to improve lameness, a better understanding of the effects of bone mineral density is warranted to identify novel therapeutic strategies. In this study, we found that housing type, age, and serum calcium level were significantly associated with bone mineral density. Any factors that can affect bone mineral density are worthy of attention. Bone mineral density increases with increasing age and reaches the maximum value in boars at the age of 43 months. Similar to humans, boar bone mineral density has a convex quadratic curve relationship with age [34]. In addition, the bone conversion makers in serum are consistent with the trend of bone mineral density.

At the same time, we also found that the bone mineral density of the Duroc boar in the large individual pen model was significantly higher than that of the Duroc boar in the individual stall model. Indeed, this finding is consistent with the results of Schenck et al. (2008), who found that exercise was more efficient to increase bone mineral density and bone strength of the humerus, radius, and tibia of sows [18]. Another interesting finding is that CTX-1, a marker of bone resorption, was lower in boars housed in the large individual pen model, and OCN, a marker of bone formation, did not differ between housing models. However, there is still limited research on these serum markers in boars and more experiments are needed to confirm the effectiveness of bone turnover markers in boars. Nevertheless, we can draw some conclusions. The structural design of large individual pens can provide more free space for the movement of boars. Although the structure design of individual stalls saves space, it limits the movement of boars. As a result, bone resorption is increased, and bone mineral density is decreased. This is similar to disuse osteoporosis, as the lack of mechanical stimulation leads to greater bone resorption than bone formation, which eventually results in decreased bone mineral density and mass [35].

Minerals are critical components of bone structure and play a significant role in maintaining overall bone health [36]. The results of our study revealed that bone mineral density increases with increasing serum Ca. This is consistent with findings of [37]. However, bone mineral density decreased with increasing serum P, in contrast with previous studies [38,39]. However, it has also been reported that there is a negative correlation between serum P and alkaline phosphatase [40]. At the same time, we also found that bone mineral density reached its maximum value when the calcium-to-phosphorus ratio was 3.2. While some research has explored the influence of the calcium-to-phosphate ratio on bone health [41,42], a consensus on the ideal ratio for optimal bone density has yet to be reached. Therefore, it is essential to conduct further experiments to examine and ascertain the effect of serum Ca and the calcium-to-phosphorus ratio on bone mineral density in boars.

## 5. Conclusions

Claw health and bone health are important influencing factors for lameness in boars. More importantly, boars with claw lesions and lower bone mineral density seem to develop lameness more readily than normal boars. Hence, reducing the prevalence of claw lesions can effectively reduce the prevalence of lameness. It is therefore important to advocate for better management and good hygienic conditions at pig farms to mitigate the prevalence of lameness. On the other hand, strong bones may prevent the occurrence of lameness. Therefore, performing suitable movements with effective management may be able to reduce the prevalence of lameness by increasing bone mineral density. Simple housing adjustments, such as increased movement space, may be able to increase the movement of boars each day in an IP model housing situation, which will increase bone formation, decrease bone resorption, and reduce the prevalence of lameness of boars.

In conclusion, this study investigated the relationship between claw lesions, bone mineral density, and lameness of boars, and uncovered the neglected problems of boar breeding management in production practice. Producers should pay more attention to the management of pig farm hygiene and appropriately increase the amount of exercise for boars. In addition, bone mineral density may be used in the future as an additional tool in lameness evaluation and prediction.

## Figures and Tables

**Table 1:** Evaluation standard of claw lesions [22].

Lesion Type			Description of Lesions	
	0 (Normal)	1 (Mild)	2 (Moderate)	3 (Severe)
Toes (TOE)	Normal	One or more toes slightly longer than normal	One or more toes significantly longer than normal	Long toes that affect gait when walking
Dew claws (DEW)	Normal	Slightly longer than normal	Claws extend to floor surface when the pig is standing	Claw is torn and/or partially or completely missing
Heel overgrowth and erosion overgrowth (HOE)	Normal	Slight overgrowth and/or erosion in soft heel tissue	Numerous cracks with obvious overgrowth and erosion	Large amount of erosion and overgrowth with cracks throughout
Heel-sole crack (HSC)	Normal	Slight separation at the juncture	Long separation at the juncture	Long and deep separation at the juncture
White line (WL)	Normal	Shallow and/or short separation along white line	Long separation along white line	Long and deep separation along white line
Cracked wall horizontal (CWH)	Normal	Hemorrhage evident, short/shallow horizontal crack in toe wall	Long but shallow horizontal crack in toe wall	Multiple or deep horizontal crack(s) in toe wall
Cracked wall vertical (CWV)	Normal	Short/shallow vertical crack in wall	Long but shallow vertical crack in wall	Multiple or deep vertical crack(s) in wall
Swelling Ankle (SWE)	Normal	Ankle with slight swelling at coronary band	Ankle with swelling at coronary band and sole	Ankle with swelling at coronary band and hole claw

**Table 2:** Data distribution of bone mineral density in experimental boars.

Group	Classification Criteria	SOS Value, m/s	n	%
Strong bone	SOS > $\bar{X}$ + SD	≥4426	75	10.15
Normal bone	$\bar{X}$ − SD ≤ SOS ≤ $\bar{X}$ + SD	3976~4425	524	70.91
Osteopenia	$\bar{X}$ − 2.5 × SD ≤ SOS < $\bar{X}$ − SD	3639~3975	128	17.32
Osteoporosis	SOS < $\bar{X}$ − 2.5 × SD	≤3638	12	1.62

**Table 3:** Potential influencing factors of bone mineral density of boars were screened by univariate logistic regression analysis.

Risk Variable	1 = Strong Bone (n = 75)	2 = Normal Bone (n = 524)	3 = Osteopenia (n = 140)	*p* Value	OR (95%CI) ^1^
**Age**					
1 = ≤12	0 (0)	165 (31.48%)	57 (40.72%)	<0.001	7.957 (1.527–2.621)
2 = 13–24	17 (22.67%) ^3^	258 (49.24%)	54 (38.57%)	<0.001	4.446 (0.982–2.002)
3 = 25–36	21 (28.00%)	51 (9.732%)	12 (8.57%)	0.169	1.559 (−0.189–1.077)
4 = ≥37 (Ref) ^2^	37 (49.33%)	50 (9.54%)	17 (12.14%)	-	-
**Body weight, Kg**					
1 = ≤200	0 (0)	110 (20.99%)	37 (26.43%)	<0.001	5.613 (1.109–2.341)
2 = 201–250	8 (10.67%)	204 (38.93%)	59 (42.14%)	<0.001	4.437 (0.921–2.059)
3 = 251–300	42 (56.00%)	167 (31.87%)	30 (21.43%)	0.121	1.554 (−0.117–0.999)
4 = ≥301 (Ref)	25 (33.33%)	43 (8.21%)	14 (10.00%)	-	-
**Housing type**					
1 = IS	7 (9.33%)	264 (50.38%)	93 (66.43%)	<0.001	3.557 (0.921–1.618)
2 = IP (Ref)	68 (90.67%)	260 (49.62%)	47 (33.57%)	-	-
**Ca, mg/dL**					
1 = ≤8	5 (6.67%)	52 (9.92%)	26 (18.57%)	0.042	1.765 (0.021–1.115)
2 = 8–11	56 (74.67%)	342 (65.27%)	80 (57.14%)	0.190	0.778 (−0.628–0.125)
3 = ≥11 (Ref)	14 (18.66%)	130 (24.81%)	34 (24.29%)	-	-
**P, mg/dL**					
1 = ≤4	16 (21.33%)	30 (5.73%)	6 (4.29%)	<0.001	0.140 (−2.743–−1.183)
2 = 4–9	59 (78.67%)	446 (85.11%)	115 (82.14%)	0.007	0.486 (−1.245–−0.197)
3 = ≥9 (Ref)	0 (0)	48 (9.16%)	19 (13.57%)	-	-
**Zn, mg/L**					
1 = ≤0.5	30 (40.00%)	207 (39.50%)	70 (50.00%)	0.080	1.374 (−0.038–0.673)
2 = 0.5–1.0	15 (20.00%)	118 (22.52%)	25 (17.86%)	0.884	1.033 (−0.397–0.461)
3 = ≥1.0 (Ref)	30 (40.00%)	199 (37.98%)	45 (32.14%)	-	-
**Mg, mg/L**					
1 = ≤18	19 (25.33%)	131 (25.00%)	35 (25.00%)	0.739	1.093 (−0.435–0.613)
2 = 18–22	48 (64.00%)	312 (59.54%)	90 (64.29%)	0.614	1.127 (−0.345–0.585)
3 = ≥22 (Ref)	8 (10.67%)	81 (15.46%)	15 (10.71%)	-	-
**Cu, mg/L**					
1 = ≤2.0	34 (45.33%)	212 (40.46%)	57 (40.72%)	0.349	0.788 (−0.737–0.260)
2 = 2.0–2.5	35 (46.67%)	243 (46.37%)	64 (45.71%)	0.415	0.715 (−0.695–0.287)
3 = ≥2.5 (Ref)	6 (8.00%)	69 (13.17%)	19 (13.57%)	-	-
**Fe, mg/L**					
1 = ≤1.0	34 (45.33%)	242 (46.18%)	76 (54.28%)	0.882	1.041 (−0.490–0.571)
2 = 1.0–2.5	37 (49.33%)	221 (42.18%)	51 (36.43%)	0.331	0.765 (−0.807–0.272)
3 = ≥2.5 (Ref)	4 (5.34%)	61 (11.64%)	13 (9.29%)	-	-
**Mn, μg/L**					
1 = 0	39 (52.00%)	335 (63.93%)	91 (65.00%)	0.207	1.323 (−0.155–0.714)
2 = 0–30	15 (20.00%)	112 (21.37%)	23 (16.43%)	0.880	1.041 (−0.482–0.562)
3 = ≥30 (Ref)	21 (28.00%)	77 (14.70%)	26 (18.57%)	-	-
**Se, μg/L**					
1 = ≤150	7 (9.33%)	145 (27.67%)	41 (29.29%)	0.241	1.357 (0.205–0.814)
2 = 150–300	56 (74.67%)	305 (58.21%)	77 (55.00%)	0.422	0.829 (−0.648–0.272)
3 = ≥300 (Ref)	12 (16.00%)	74 (14.12%)	22 (15.71%)	-	-
**Pb, μg/L**					
1 = 0	54 (72.00%)	402 (76.72%)	109 (77.86%)	0.397	1.175 (−0.211–0.532)
2 = >0 (Ref)	21 (28.00%)	122 (23.28%)	31 (22.14%)	-	-
**Cd, μg/L**					
1 = 0	64 (85.33%)	470 (89.69%)	122 (87.14%)	0.991	1.003 (−0.493–0.499)
2 = >0 (Ref)	11 (14.67%)	54 (10.31%)	18 (12.86%)	-	-

^1^ OR = odds ratio; CI = confidence interval. ^2^ Ref = reference. ^3^ 17 (22.67%): outside the parentheses is the sample size, n = 17; the proportion of the sample size in the group is shown in parentheses, which is 22.67%.

**Table 4:** The results of the multivariate ordered logistic regression model analysis of the factors affecting bone mineral density in boars.

Risk Variable	Estimate	SE ^1^	Odds Ratio	95% CI ^2^		*p* Value
				Lower 95%	Upper 95%	
Intercert 1	−0.943	0.449	-	−1.822	−0.063	0.036
Intercert 2	3.314	0.474	-	2.386	4.242	<0.001
**Housing type**						
1 = IS	1.259	0.243	3.522	0.784	1.734	<0.001
2 = IP (Ref) ^3^	0	0	0	0	0	-
**Age, months**						
1 = ≤12	1.310	0.462	3.706	0.403	2.216	0.005
2 = 13–24	1.774	0.385	5.894	1.021	2.528	<0.001
3 = 25–36	0.833	0.394	2.300	0.061	1.604	0.034
4 = ≥37 (Ref)	0	0	0	0	0	-
**Body weight, Kg**						
1 = ≤200	−0.156	0.503	0.856	−1.141	0.829	0.756
2 = 201–250	−0.127	0.435	0.881	−0.980	0.725	0.770
3 = 251–300	−0.342	0.377	0.710	−1.081	0.397	0.364
4 = ≥301 (Ref)	0	0	0	0	0	-
**Ca, mg/dL**						
1 = ≤8	0.636	0.300	1.889	0.049	1.224	0.034
2 = 8–11	−0.233	0.199	0.792	−0.624	0.157	0.241
3 = ≥11 (Ref)	0	0	0	0	0	-
**P, mg/dL**						
1 = ≤4	−0.753	0.449	0.471	−1.632	0.126	0.093
2 = 4–9	−0.180	0.294	0.835	−0.757	0.396	0.540
3 = ≥9 (Ref)	0	0	0	0	0	-
**Zn, mg/L**						
1 = ≤0.5	0.336	0.191	1.400	−0.038	0.709	0.078
2 = 0.5–1.0	0.037	0.224	1.038	−0.403	0.477	0.868
3 = ≥1.0 (Ref)	0	0	0	0	0	-

^1^ SE = standard error. ^2^ CI = confidence interval. ^3^ Ref = reference.

## Data Availability

Not applicable.
